# The cost of treating an acute ischaemic stroke event and follow-up at a teaching hospital in Malaysia: a Casemix costing analysis

**DOI:** 10.1186/1472-6963-12-S1-P6

**Published:** 2012-11-21

**Authors:** FAA Aznida, Nor MN Azlin, MN Amrizal, S Saperi, SM Aljunid

**Affiliations:** 1 United Nations University International Institute for Global Health, Kuala Lumpur, Malaysia; 2 University Kebangsaan Malaysia, Kuala Lumpur, Malaysia

## Introduction

Stroke is a continuum spectrum of complications related to the cerebrovascular event. Economic evaluation usually estimates costs incurred during acute management phase. Costs for managing post stroke patients at Specialist clinic setting and outpatient rehabilitation are of interest for developing countries which largely provide this type of service. We aim to determine costs for treating acute ischaemic stroke and subsequent follow-up after discharge at a tertiary teaching hospital.

## Methods

Cost analysis was done via top down costing and from the healthcare providers’ perspective. Casemix database was used to collect data on consecutive admissions for acute stroke events based on severity for period of January to December 2010. Patients’ follow-up visits to the Specialists’ Outpatient clinics and stroke rehabilitation were traced through HIS to facilitate cost aggregation, as this is not generated by the Casemix system. MY DRG codes were referred to in estimating cost at all stages of stroke care. Codes for minor and moderate stroke were combined to represent minor stroke category.

## Results

The average length of stay in hospital for acute major stroke was 9.8 (SD7.1) days while for minor stroke 3.6 (SD1.6) days. In the 3 month period after the acute event, an average of 2 visits were made to the Specialist outpatient clinic for both the minor and major stroke types. For outpatient rehabilitation, patients attended an average of 10 and 15 sessions for minor and major stroke severity respectively over a six-month period. The cost for treating a patient with acute major stroke per admission was MYR 9000 (SD6569). For a minor stroke the cost was MYR 3353 (SD 1444). In the Outpatient setting, the cost per visit to Specialist clinic was RM103 and the cost of rehabilitation was RM43 per patient per session irrespective of stroke severity.

## Conclusion

The cost for treating an acute ischaemic stroke event is substantial in Malaysia. Major stroke consumes three times as much medical resource than a minor stroke. Primary and secondary prevention measures should be strengthened to reduce stroke prevalence and incidence in view of substantial treatment costs. Reasons for poor utilization of post-stroke treatment warrant further studies.

